# Understanding the Role of the Diagnostic ‘Reflex’ in the Elimination of Human African Trypanosomiasis

**DOI:** 10.3390/tropicalmed5020052

**Published:** 2020-04-01

**Authors:** Jennifer J. Palmer, Caroline Jones, Elizeous I. Surur, Ann H. Kelly

**Affiliations:** 1 Department of Global Health & Development, London School of Hygiene & Tropical Medicine, 15–17 Tavistock Place, London WC1H 9SH, UK; 2 Centre of African Studies, University of Edinburgh, 15a George Square, Edinburgh EH8 9LD, UK; 3 Kemri-Wellcome Trust Research Programme, P.O. Box 230, Kilifi 80108, Kenya; cjones@kemri-wellcome.org; 4 Independent consultant, Juba, South Sudan; elizeous@gmail.com; 5 Department of Global Health & Social Medicine, King’s College London, 30 Aldwych, London WC2B 4BG, UK; ann.kelly@kcl.ac.uk

**Keywords:** South Sudan, human African trypanosomiasis, diagnosis, symptoms, treatment-seeking, case detection, elimination, embodiment, expertise, serendipity

## Abstract

To successfully eliminate human African trypanosomiasis (HAT), healthcare workers (HCWs) must maintain their diagnostic acuity to identify cases as the disease becomes rarer. HAT experts refer to this concept as a ‘reflex’ which incorporates the idea that diagnostic expertise, particularly skills involved in recognising which patients should be tested, comes from embodied knowledge, accrued through practice. We investigated diagnostic pathways in the detection of 32 symptomatic HAT patients in South Sudan and found that this ‘reflex’ was not confined to HCWs. Indeed, lay people suggested patients test for HAT in more than half of cases using similar practices to HCWs, highlighting the importance of the expertise present in disease-affected communities. Three typologies of diagnostic practice characterised patients’ detection: ‘syndromic suspicion’, which closely resembled the idea of an expert diagnostic reflex, as well as ‘pragmatic testing’ and ‘serendipitous detection’, which depended on diagnostic expertise embedded in hospital and lay social structures when HAT-specific suspicion was ambivalent or even absent. As we approach elimination, health systems should embrace both expert and non-expert forms of diagnostic practice that can lead to detection. Supporting multidimensional access to HAT tests will be vital for HCWs and lay people to practice diagnosis and develop their expertise.

## 1. Background

A key area of concern in today’s drive towards elimination of human African trypanosomiasis (HAT)—a fatal but curable infectious disease also known as sleeping sickness—is whether healthcare workers (HCWs) in frontline facilities and populations in endemic areas have the necessary “reflex” to suspect HAT when they encounter someone showing characteristics of the disease [1] (p. 36). While important for any elimination campaign, for HAT, this preoccupation with peoples’ familiarity with the disease’s clinical presentation is an artefact of the particular challenges posed by its diagnosis. 

With a variable symptom presentation affecting all parts of the body including the patient’s mental capacity, descriptions of HAT have confounded clinical characterization for centuries [2]. Found only in scattered pockets of rural countryside in sub-Saharan Africa, HAT continues to be written about in medical textbooks and infectious disease case reports emphasising its exotic and mysterious character. HAT tends to be described through long lists of strange symptoms, from intermittent fevers to cardiac features, endocrine dysfunction, fertility problems, altered gait and tremors, uncontrollable episodes of sleep, lassitude, hallucinations and excessive sexual impulses [3,4], punctuated with authors’ personal observations about other, less tangible ways to recognize the disease such as the “sad or strangely expressionless face” of HAT [5] (p. 150), [6]. “Owing to the many clinical variations of sleeping sickness”, experts write, “it is difficult to describe a ’typical’ case of the disease” [7] (p. 1310). Provided clinicians have access to the laboratory infrastructure necessary to identify the parasite, “[o]nce thought of, it is not usually a difficult diagnosis to confirm” [8] (p. 681). However, given that HAT infects only 1–2% of a population during outbreaks and is even more rare in places where elimination campaigns are underway [9], both the clinical and laboratory capacity, generally understood as a prerequisite for diagnosis, tend to be limited.

Moreover, ‘active screening’, the main method adopted by HAT programmes over the last century to detect patients with the most common, *Trypanosoma brucei gambiense* form of the disease, enables programmes to avoid having to consider the best ways to identify cases through clinical questioning. By assembling all people considered at risk of harbouring the infection in an area and asking them to submit a blood sample for analysis, the approach relies on laboratory technology and decision-making algorithms to detect disease rather than direct communication with sick people about their illnesses.

As cases of HAT recede in many areas of Africa today, this more costly active approach to case detection and treatment has given way to ‘passive’ case detection approaches which test only people with suggestive symptoms and rely on cases being detected during peoples’ routine interactions with HCWs in health facilities. The recent development of rapid diagnostic tests (RDTs) for HAT, moreover, has enabled passive detection strategies to be reformed [10]. As with other RDTs, their ease of use means the first screening test in a step-wise algorithm can be taken out of hospital laboratories into primary care settings, broadening the pool of diagnosers able to test patients when they suspect disease.

This shift towards passive detection has stimulated debate in policy circles on how best to engage with the complexities of HAT case presentation and the challenges these pose for detecting the disease. The decision-making processes of HCWs have become a major point of focus. The ‘HAT reflex’ is a part of this new push towards cultivating frontline providers’ syndromic familiarity—also referred to as an “index of suspicion” [11] (p. 192)—which clinicians intimately involved in HAT control say is critical for triggering the HAT reflex.

The importance of the HAT reflex gains amplitude in an elimination setting. The scarcity of cases is believed to pose a particular challenge for passive case detection as HCWs lose their diagnostic expertise due to infrequent contact with the disease. According to this argument, maintaining diagnostic acuity or ‘reflexes’ is a matter of habitual practice, whether practiced in the clinic among patients or in the lab dealing with samples. This position is captured by a recent World Health Organisation (WHO) guidance document on HAT elimination:

“In health centres where transmission of the disease is high, staff deal with gambiense HAT frequently, and clinical [syndromic] suspicion and laboratory work are usually done correctly; but in areas of low transmission, health workers lack the reflex to think about this disease, which has become uncommon, laboratory staff are unfamiliar with HAT tests”.

[1] (p. 36)

Contact with the disease is also conceptualised as an important prompt for populations living in an endemic area to think of HAT as an individual risk and a collective public health priority, though medical language such as a ‘low index of suspicion’ is not explicitly used. Decreasing fear of the disease is often cited as an explanation for decreasing participation in active screening campaigns over time [12,13,14,15].

The possibilities of cultivating a diagnostic reflex for HAT among HCWs with minimal clinical training and where health infrastructure is limited provides a promising point of departure to support passive case detection approaches for elimination. However, despite the almost common-sensical importance of enhancing diagnostic acuity, how a reflex operates in practice and how it might be deployed within a programmatic setting remains unclear.

Granular explorations of how HAT diagnosis actually happens in a passive detection context have also been limited. Indeed, despite its contribution to disease control over the last several decades [16], there has been remarkably little research on passive case detection, in general [12]. This empirical gap is perhaps particularly surprising considering the role this approach is expected to play in achieving elimination of HAT in the coming decade.

In this paper, we seek to address what we see as HAT experts’ interlinked concerns about a reduction in HCWs’ and lay peoples’ ability to diagnose HAT in the era of elimination. We ask, what exactly is a ‘diagnostic reflex’ and could endemic area health systems inculcate one for HAT? To answer these questions, we begin by introducing some key theories about medical diagnosis and case detection. We then present an in-depth study of the diagnostic practices involved in the successful passive detection of 32 cases from a hospital-based HAT programme in South Sudan where HCWs and the public still had relatively frequent contact with the disease. In so doing, we reflect upon what sorts of knowledge, material and social engagements facilitated the exercising of a HAT reflex in this setting and expand on how a HAT reflex could be conceptualised to work in practice. In conclusion, we suggest how such practices may potentially be translated to other settings in preparation for elimination.

### The Diagnostic Reflex in Theory

The term, ‘reflex’, tends to refer to automatic behaviour. Like a neurological reflex which responds to stimuli automatically along pathways that bypass the brain’s cognitive reasoning functions, the HAT diagnostic reflex suggests an almost immediate recognition of HAT when presented with a clinical case of the disease. As alluded to in the WHO passage, the skill is not learned through guidelines and training alone, but rather acquired through regular contact with patients and disease—or what medical sociologists term, ‘embodied knowledge’.

Practicing medicine combines aspects of both abstract “embrained” and practical “embodied” knowledge [17,18] (p. 490). Whereas embrained knowledge is explicit, objective and depends on abstract cognitive reasoning, embodied knowledge is tacit, intuitive and builds upon ‘bodily’ or practical experience in a particular context. Embodied knowledge is automatic; its generation and application does not need to be processed through conscious decision-making. One example is the way HCWs learn to gauge a fever by putting their hand to a patient’s forehead through cultivating a sensitivity to body temperature.

The transformation of that sense into a ‘symptom’ is both a cognitive and cultural process, involving integrating medical knowledge on one hand, and judgements about what constitutes normal and abnormal on the other [19,20,21]. The clinical training HCWs undertake contextualizes a sensibility of illness within biomedical theories of etiology and pathogenesis, enabling them to detect a HAT infection, for example, through the ‘look’ on a patient face. Their diagnostic expertise, in other words, develops through an integration of formal, codified categories with the social norms and habits associated with particular healthcare settings [22]. This ability to recognise disease is iteratively shaped through routine interactions with patients and colleagues, shared within groups and embedded in working cultures, becoming gradually more automatic and reflex-like through collaborative practice [18,23,24,25,26,27].

An example of this process of integrating embrained and embodied knowledge is the HCW’s use of storytelling. To deal with the diagnostic uncertainty they face routinely, HCWs develop internal narrative scripts or templates based on a personal bank of experience with similar patients. Diagnostic scripts help a clinician look backward and forward simultaneously, (re-)telling a patient’s syndromic history in a way that fits the narrative and predicts the future prognosis. These stories are shaped by what HCWs think they can do for the patient based on past precedent and by the rules and routines within their healthcare facility using “clinical judgement” [28] (p. 873). Given that diagnostic instruments, such as a microscope or serological agglutination test, do not create meaning independently of the practitioners who use them [29], scripts are what allow HCWs to place test results (as well as symptoms and other forms of evidence) in context. By providing a shortcut through cognitive processes, scripts quickly help a HCW settle on a pathway for action, but can also constrain available options since “knowing where to look strongly affects what clinicians can find” [28] (p. 875).

The diagnostic reflex is not only the province of medical professionals, however. Lay people also routinely rely on embodied knowledge to monitor symptoms and detect illness both before and after ‘diagnosis’ by a healthcare provider as well as during and after treatment [19,30]. Like that practiced by clinicians, this monitoring involves evaluating different forms of evidence, such as the observations of others or the results of blood tests, which prompts action such as visiting another health facility.

Like clinicians’ use of scripts to proscribe practical actions, choosing where to seek a diagnosis can also be seen as involving diagnostic expertise developed through practice. Accessing diagnostic services within a health system requires substantial practical navigational expertise from patients, their families and other lay people within their social networks [31,32]. Sometimes the shortest or most straightforward route to successful care is dependent upon belonging to a social network with established links to clinical providers [33]. Information about HAT symptoms [19] and HAT service availability [34] circulates through cultural and linguistic networks within ethnic and migrant groups which can have widely different knowledge of and experiences with hospital care [34,35].

Seeking HCW expertise when people are uncertain about the meaning of symptoms is, moreover, often only one reason a patient visits a health facility. Patients may access services when they already have a good idea of what the problem is or, on some occasions, to try out new tests and compare results from the facilities where they have been tested previously [31,36]. Patients can prompt HCWs to pursue particular diagnoses [37] or submit to provider-led testing as a means of assisting communication about their illness with HCWs in a way that is acceptable to clinicians [38]. Patient-led testing (also known as voluntary or self-testing), can be seen as a healthcare coping strategy used by patients who have had negative interactions with HCWs in the past or as the behaviour of modern, information-savvy patients fostered in programmes that encourage people to take individual responsibility for their health [31,34,39].

In short, locating the HAT reflex as something that happens solely in HCWs’ clinical encounters risks conflating passive case detection with simplistic ideas about treatment-seeking where responsibility for diagnosis is neatly divided between lay people and medical experts, with everything occurring before a patient’s first contact with health services under the control of lay peoples’ decisions and actions and everything after the responsibility of HCWs [33,40]. Passive case detection, after all, is only a ‘passive’ act from the perspective of a programme manager considering the additional effort and costs required to implement active screening [41]. Using such terminology tends to obscure the key role of patients and other lay people through the diagnostic process. To arrive at a diagnosis and successfully identify a case requires a great deal of patient engagement with the health system, as they proactively seek out treatment for their illness.

## 2. Methods

We collected detailed stories of successful case detection about patients identified by the HAT programme in Nimule, South Sudan over eight months in 2008–2009. Our recent work on how diagnostic technologies are integrated into global health research and response have encouraged us to contextualise these insights and reflect on this empirical material anew [42,43].

### 2.1. Setting

At the time of fieldwork, which happened before the global roll-out of RDTs for HAT, Nimule Hospital supported by the non-governmental organisation, Merlin, was one of only eight sites in the country which could offer HAT detection and treatment. HAT services had been available here and through intermittent active screening campaigns since 2005. During this period, the prevalence of *gambiense* sleeping sickness was around 1% in the public and 2.8% among patients presenting to the HAT service [34]. As the only functioning hospital serving a county population of around 170,000 [44], Nimule hospital was an important focus of general healthcare activity for people living in and far from the town. The HAT service itself was very popular, with 13,815 people screened passively over a five-year period from 2005 to 2010 [34].

HAT patients were diagnosed in the laboratory using a multi-stepped screening algorithm based on the card agglutination test for trypanosomiasis (CATT) (see Supplemental File S1). Like most diagnostic work performed in the laboratory, HAT practices were codified in a hospital protocol based on national programme and World Health Organisation guidance.

At the end of 2009, hospital managers began to draft symptom-based flow-charts for a variety of common patient presentations to aid diagnostic practice in the outpatient department. Their desire for formal guidance on HAT prompted a subsequent study by us on the predictive value of patients’ presenting symptoms [45]. For the majority of fieldwork, however, written or visual materials that could have contributed to a material-culture of syndromic diagnosis in the hospital such as algorithms, guidelines and posters, were largely absent. Medical doctors and clinical officers were simply asked to use their discretion to decide when to order a HAT test.

Ordering a test involved requesting lab staff collect a venous blood sample from inpatients in the wards or sending outpatients to a specially demarcated section of the lab for HAT. This section had a separate entrance used to manage screening the large numbers of people who wanted a HAT test when the programme was first introduced and whose demand could not be met by the limited active screening services the programme could offer. Members of the public could request a test for themselves here without referral by a HCW or first discuss their suspicion during an outpatient consultation.

### 2.2. Data Collection and Analysis

We constructed case studies around 32 patients who were detected at the hospital. Twenty-six were new cases, six had been dismissed by the programme from further follow-up (five who had been treated for HAT 3–7 years earlier and one who had temporarily been considered a serological suspect). These patients represented 61% of cases identified passively during the study period. Between one and three patients per week were followed depending on admission patterns and research team capacity, with patients selected to achieve maximum variation of demographic characteristics (14 females and 18 males aged 11–65 years old, including 16 Dinka, 11 Madi and 1 each of Acholi, Kakwa, Lotuko, Lolubo and mixed Lango-Acholi ethnicities). Nearly all were in the final (second) stage, defined as when parasites can be found in the brain or spinal fluid (27 in stage 2, two in stage 1; three others died between the time when infection was confirmed and disease staging could be carried out).

Case studies were initiated as soon as the research team was notified that a case had been detected, beginning with interviews with hospital staff involved in diagnosis. Research team members were trained in HAT screening procedures and embedded in the lab during weekday mornings to observe many patients’ earliest interactions with the programme; team members also observed all medical ward admission interviews required for the initiation of HAT treatment. In-depth research interviews were then conducted with patients or their guardians (if the patient was under 18 years old or had a cognitive impairment that made it difficult to participate fully) on the first or second day of treatment. Extensive field notes were also collected during the patient’s hospital stay to capture additional information from patients, their families and friends, health staff and medical observations in patient files. 

We explored how a “HAT reflex” comes about by asking patients and family members to narrate their health-seeking activities over the last several years in relation to symptoms that motivated the patient’s presentation at the hospital. We asked HCWs (doctors, clinical officers, nurses and laboratory technicians) to recall details of their interaction with patients. Bearing in mind the contributions of lay people to diagnosis and the potentially habitual and iterative nature of HAT diagnostic practices, the focus of enquiries was to identify the critical events that occurred immediately before HAT was identified in the lab, particularly who suggested the patient be tested for HAT and why, considering a range of syndromic experiences and material and social contextual circumstances [46]. Based on analysis of these critical events, qualitative typologies were identified of the diagnostic practices which led to successful detection. 

Typologies were initially analysed separately according to the category of person who suggested the diagnosis (the ‘diagnoser’, either a lay person without biomedical training who advised the patient to seek a HAT test or a HCW who ordered or performed one without a specific prompt from the patient) and brought together in the final presentation of results which focused on understanding the interaction of knowledge and habit in context (see Supplemental File S2). In some key cases, the diagnosis was arrived at in the absence of a decision by any individual who might be classified as a diagnose [47]. What we describe as ‘serendipitous’ cases, hinged entirely on a series of contingent events which were often set in motion by a willingness or openness to the possibilities of the medical diagnostic process to arrive at a cause for the illness.

Further details of the interviewing and analysis process, including how we addressed some anticipated limitations of interviewing people about the treatment-seeking and diagnostic process are available in Supplemental File S3. Interviewees’ detailed experiences of sense-making in relation to HAT symptoms is reported elsewhere [19]. Additional data on patients’ treatment-seeking characteristics including the length of time spent seeking treatment and the number of health facilities visited before detection, with methods used to collect it, are available in Supplemental File S4. The alternative diagnoses patients received in relation to their presenting symptoms are available in Supplemental File S5.

All subjects gave their informed consent for inclusion before they participated in the study. The study was conducted in accordance with the Declaration of Helsinki, and the protocol was approved by the London School of Hygiene and Tropical Medicine’s ethical review committee (project ID 5507) and the Ministry of Health, Government (now Republic) of South Sudan.

## 3. Findings

Three typologies of diagnostic practice explained what led patients to be diagnosed with HAT: ‘syndromic suspicion’, ‘pragmatic testing’ and ‘serendipitous detection’ (see Box 1 for definitions of each).

Box 1 **Definitions of diagnostic practice typologies which led to successful human African trypanosomiasis (HAT) case detection, highlighting the interplay of knowledge and habit.**

*Syndromic suspicion:* HAT test suggested or ordered based on the diagnosing person’s recognition of HAT symptoms. The process of symptom recognition was enabled by embodied knowledge of symptoms accrued through direct exposure to the disease. Closely resembled the idea of an expert HAT reflex.

*Pragmatic testing:* HAT test suggested or ordered during a process where the diagnosing person was concerned with ‘trying’ tests for multiple diseases, but their specific suspicion of HAT was ambivalent. While HAT testing was not prompted by an expert syndromic reflex, it was supported through practical knowledge of HAT tests and habitual testing behaviour.

*Serendipitous detection:* HAT test performed without any specific suspicion of HAT or even a request to test from an individual, with detection based on expertise embedded in the habitual testing practices of the hospital or lay social network.

**Figure 1:**
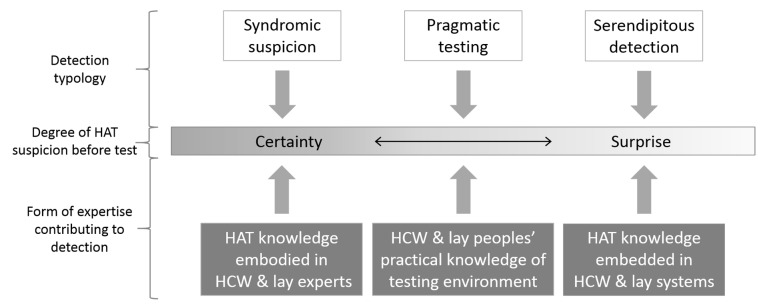
The gradient of certainty versus surprise along which human African trypanosomiasis (HAT) passive case detection happened, indicating the form of expertise which led to diagnosis. (HCW: healthcare worker).

A key characteristic which shaped and differentiated these typologies was the degree to which people involved in the case depended on syndromic logic to guide testing decisions. In cases assigned a syndromic suspicion typology, HAT testing decisions appeared deliberative, based on considerations about whether HAT could explain observed symptoms, and often drawing on considerable embodied knowledge suggesting familiarity with the disease presentation. These cases resembled most closely the idea of the HAT reflex in expert discourses. In cases identified through pragmatic testing, HAT-specific suspicion was more ambivalent, emerging only when syndromic suspicion for more common infections failed. Drawing on diagnosers’ knowledge of the local testing environment, the logic involved was pragmatic rather than symptomatic. HAT tests were requested to identify new treatment options without necessarily settling on disease labels—a diagnostic openness that enabled a more pragmatic course of therapeutic orientation [48]. Finally, in cases involving serendipitous detection, there appeared to be no specific suspicion of HAT at all and case detection came as a surprise. In these circumstances, no true ‘diagnoser’ was involved and it was the diagnostic expertise embedded within hospital processes and social networks coupled with peoples’ openness towards these institutions which enabled detection in the absence of a HAT reflex in individuals. The typologies therefore suggested that HAT diagnosis happened along a gradient of degrees of suspicion, ranging from diagnostic certainty to surprise, and relying on the diagnostic expertise both embodied in individuals and embedded in systems (Figure 1).

The case studies also highlighted how HAT diagnostic practice was distributed beyond HCWs and healthcare environments to include patients and other lay populations within a wider diagnostic or case detection *system*. Most cases in the series (19/32) were detected after a lay person decided or advised a patient to test for HAT. These included neighbours, teachers, relatives living with patients or visiting on short trips, as well as patients themselves. HCWs were responsible for detecting the remainder (13/32 including 10 from Nimule Hospital and one each from primary healthcare facilities, military barracks and other hospitals), through formal consultations and in informal social interactions with patients. The diagnostic practices lay people engaged in were very similar to HCWs’ (Table 1). Both HCWs and lay people successfully diagnosed HAT with an embodied syndromic reflex (nine cases identified by each type of diagnoser). They both also resorted to HAT testing through the pragmatic approach when no syndromic diagnosis was likely, though this was more common in cases where testing was initiated by lay people (eight by lay people vs. two by HCWs). Finally, the systems created by each group also supported HAT diagnosis, whether this was a social network created by lay people to communicate useful information about test availability or the processes and practices instituted by HCWs to manage patients and samples within the hospital. We turn to illustrations of the specific typologies next.

### 3.1. Syndromic Suspicion

Cases detected according to this typology closely resembled the idea of the HAT reflex in expert discourses. In the following sections we explore three concepts which help further characterise the HAT reflex: it involves practical, embodied knowledge that emerges from HCW experience with HAT and other diseases and which suits the diagnostic resources available; the habitual prompts or internal narrative ‘scripts’ that HCWs use to identify HAT frame abnormal behavioural and neurological symptoms as biomedical; and lay people also have a HAT reflex.

#### 3.1.1. Use of Embodied Knowledge

A doctor working on the medical ward recalled how he sensed something strange in the “vacant expression” of a severely ill and febrile pregnant woman. As malaria affects pregnancy so severely, he immediately began her on treatment for cerebral malaria. However, his practical, embodied understanding of what he would expect a critically ill malaria patient to look like prompted him to explore alternatives. He decided that HAT should be included in his differential diagnosis “because these [malaria and HAT] are the diseases that can affect the central nervous system” and ordered both a malaria and a HAT test, successfully identifying the co-infections and her as a case of HAT.

The doctor’s explanation of how he identified the differential diagnoses exemplified HCWs’ en-skilled understandings of disease, which led them to suspect HAT when faced with complex forms of illness. In this instance, the doctor, who had lived most of his life in the area and managed many HAT patients before, drew from this contextual knowledge on how to manage patients with severe symptoms impairing consciousness which embedded the diagnostic possibility of HAT alongside more common diseases. Given the acute, febrile nature of the patient’s condition, the doctor felt an infectious disease was most likely and some of the easiest diseases to test for in this setting (as opposed to, say, bacterial meningitis), were malaria and HAT. Before the test had even been performed, the neurological nature of the condition led the doctor to consider HAT, helping him to make sense of his earlier subtle observation of the patient’s vacant expression in light of what he already knew about the disease and label it, prompting syndromic recognition of HAT.

#### 3.1.2. Biomedical Framing

The kinds of symptoms which prompted a HAT reflex among HCW diagnosers were associated with two main narrative scripts: severe illness impairing consciousness as already described and severe mental confusion including hallucinations. Examples of presentations in cases which fit these scripts included intense nightmares in a child, violent paranoia in a woman, convulsions in a man, indications of partial or mild paralysis as well as various degrees of excessive sleeping.

Stemming from their biomedical training, the narrative scripts HCWs developed and used were specifically biomedical, which enabled personnel to suspect HAT when those around patients did not see a medical illness at all. For example, one day in the medical ward, a woman arrived with an abdominal knife wound she had inflicted on herself and a letter from the police. It read:

To the Medical Officer, Merlin [Nimule] hospital. The above named woman reported to police. She knifed herself alone when her mind got confused. Please help to treat her.

The woman was involved in brewing alcohol for neighbours who were commonly drunk around her and when she became irrationally violent towards them, they taunted her into turning a knife on herself. Unlike the knife wound which needed acute care, family members did not consider this woman’s behaviour as symptomatic of an illness that would require hospital consultation itself. Following the script for common causes of mental confusion, however, since the woman did not have a long history of mental illness the clinical officer suspected the event could be explained by a paranoid hallucination induced by an infectious disease such as HAT. Neighbours initially took the woman to the police, reasoning that an expert in security was needed since they framed the problem as dangerous behaviour. It was an expert in medicine, however, who interpreted the behaviour as a medical symptom in need of a biomedical diagnosis which led to her case detection.

#### 3.1.3. Lay Expertise

Our data also suggested that the idea of the HAT reflex should be extended to recognise the expertise of lay people in HAT diagnosis with their own local, social forms of knowledge.

Whereas HCWs gained formal knowledge from preclinical and in-service trainings and practical experience working in HAT screening and treatment programmes, lay people acquired their knowledge of HAT symptoms from non-medical sources which were nevertheless robust. The local population’s long experience of living with the disease and interacting with active and passive screening services enabled the development of detailed popular syndromic discourses about HAT as well as practical experience observing patients become sick [19].

In stories about HAT testing initiated by lay people, the incorporation of abstract HAT knowledge was evident from diagnosers’ recollections that their suspicion of HAT was prompted by hallmark signs. The husband of one patient, for example, discussed enlarged lymph nodes as such a characteristic symptom of HAT that, when they noticed it, his family knew a HAT test was needed to confirm their suspicion and secure her treatment. He said:

She was developing lymph nodes around her neck, so some of our family members said why don’t I try a sleeping sickness test? If this symptom had appeared earlier, before going to Uganda, I would not have bothered looking for other treatment, I would have come direct for a sleeping sickness test.

A familiar feeling of body pain and weakness while doing agriculture work is the sort of practical HAT knowledge which prompted another patient who had previously been treated to return to the hospital for a HAT test. She recalled, “when it started like that again, I knew nothing else apart from sleeping sickness. That is why even if I was told controls [post-treatment follow-up tests] were over, I didn’t go to look for other tests”.

A mother’s story of how she suspected HAT in her daughter illustrates that popular syndromic knowledge was also incorporated into narrative scripts used by lay people, similarly to HCWs. She said:

I began to know that she is sick because whenever she came from school she would complain of headache [...] if she failed to get food [...] she would do nothing but sit and begin to close her eyes [...] so I decided, let her come for this test [49] [...] It is good that the sickness is found in an early stage so [...] the child will survive after treatment [...] I heard of a person with sleeping sickness from Uganda. [She] was so sick whereby she had some swellings on her face and some parts of the body and her child was the same [...] this child died.

Reflecting on the ‘tools’ most available to lay people outside of health facilities, the mother used her senses to observe her daughters’ symptoms in the context of domestic life. She empathetically observed the patient’s head pains, hunger and sleepiness which were symptoms commonly known to occur in HAT [19]. As “there is no sickness which causes people to eat a lot other than sleeping sickness”, a symptom script about eating too much prompted the mother to consider HAT, and the other symptoms supported this. Disease-specific scripts tell the story of a disease’s prognosis as well as what can practically be done [28], so once the mother thought of HAT, which had another script of its own, she knew to monitor for swelling. Combining biomedical and ethnophysiological concepts, ‘swelling’ in HAT was associated with a serious prognosis because of a homologous association with illness progression in magical poisoning [19]. Fear of her daughter developing this symptom was what prompted the mother to take her to Nimule hospital for a HAT test where she was identified as a case.

Lay diagnosers also clearly possessed agency to ensure diagnostic work was done, driven by their syndromic suspicions. This was particularly true of stories about patients who had to work against the habits and rules of the lab’s self-testing service for detection to happen. Knowing that patients with negative CATT screening results could not be retested within three months, for example, the families of two cases (both children), returned at quarterly intervals for testing until the children were confirmed. Previously treated patients who had completed their two years of follow-up testing, such as the woman with a familiar feeling of body pain and weakness, also sometimes reported that they were not “allowed” to test for HAT after this point [50]. When she returned for a test, she was twice refused. On the third visit, the patient said she decided to “just keep quiet” and resorted to a small deception by posing as a new tester in the HAT lab queue. This enabled her to be taken through the full diagnostic algorithm used in the lab and confirmed as a case, again. Such stories demonstrated the surprising amount of tenacity involved for some lay people to access tests and achieve test results which matched what they sensed in patient bodies and communicate this to HCWs who could admit them for treatment. Like HCW diagnosers, these lay diagnosers used HAT tests in concerted ways to confirm their suspicions of HAT, unlike in cases belonging to the pragmatic testing typology, discussed next.

### 3.2. Pragmatic Testing

In diagnostic stories which followed a pragmatic testing typology, knowledge and habit enabled HAT case detection in a way that did not require the people involved to possess strong syndromic suspicion. The following case is illustrative.

The father of a young girl had consulted staff at local clinics and drug shops several times for her recurrent fevers and stomach pains. She was treated sequentially for schistosomiasis, typhoid and brucellosis but she continued to be ill. The patient’s father then noticed she was sleeping a lot and that her skin was getting lighter which suggested that she was getting swollen, so he wondered if she could have worms again, or possibly HAT. When he took her to Nimule Hospital to discuss his suspicions, a HCW in the outpatient department enquired about additional HAT symptoms and noted that the patient was also disoriented, incontinent and had difficulty walking. This led the HCW to strongly suspect HAT and he agreed to test the girl for it. After the infection was identified in the lab, her father was more convinced that HAT explained the symptoms but said he would remain sceptical until she was symptom-free. He explained this ambivalent suspicion of HAT as follows:

Because she is always feeling sleepy, I said [...] maybe it is sleeping sickness which is causing her those other pains [head and muscle aches] [...] since she was treated for those other diseases and didn’t recover well. Now I would like to see if it [the correct diagnosis] is sleeping sickness or if there is more pain after treatment for sleeping sickness.

Although a suspicion of HAT on syndromic grounds seemed highly justified to the HCW who was consulted, the lay person’s decision-making which led to successful detection depended not only on the father having some syndromic awareness of HAT, but also his willingness to opportunistically try new tests and treatments to identify practical ways to help his daughter.

In contrast to the tenacity involved in accessing HAT tests to confirm syndromic suspicions described in the last typology, in cases characterised by pragmatic testing, energy directed towards HAT testing tended to be obscured by care-seeking for other common infections. In some cases, there was even someone within the patient’s therapy management group who recommended a HAT test based on what appeared to be a (syndromic) HAT reflex, but several months nevertheless elapsed before the patient was brought for a test during which the patient received other forms of care. In these situations, the HAT suspicion along with other forms of evidence and circumstantial criteria appeared to get filtered through a decision-maker who ultimately influenced the trajectory of care-seeking and the diagnostic practices pursued (or not).

In the case of the father seeking advice about worms and/or HAT, as well as many others assigned a pragmatic testing typology, finding another test to help explain unresolved symptoms was the narrative focus of peoples’ stories, rather than HAT, itself. Recounting how they decided to bring a patient to the hospital, family members might say, for example, “The only test that I forgot to take her for was this sleeping sickness test” or “only sleeping sickness testing and treatment is what she had not done”.

As language like “forgetting to test for HAT” would suggest, many people in Nimule had practical experience of HAT tests through exposure to active and passive screening services. Information about HAT tests also circulated through announcements about changes to health services shared at migration meetings, church activities and social events, as well as informal conversations among people enquiring about tests from neighbours, at pharmacies, and within hospital waiting areas [34]. HAT tests contributed to a culture of pragmatic testing that was a commonplace and important health-seeking strategy which helped people manage illness and uncertainty in Nimule. While HAT was usually considered an unlikely diagnosis, people nevertheless arrived at it when syndromic suspicion for more common infections failed through knowledge of the HAT test. Diagnosers—or ‘decision-makers’, for want of a better word—did not need to commit to a HAT suspicion before (or even after) the HAT test was performed. ‘Trying’ a HAT test, as patients typically put it, involved settling on a pathway for action but not necessarily a ‘diagnosis’. It simply required an openness to various diagnostic options. Thus, it was peoples’ practical knowledge and habitual use of HAT tests and other lab services, rather than HAT symptoms, that enabled this sort of detection.

HCWs also pursued HAT testing pragmatically when their other interventions were not working. Faced with a patient experiencing convulsions and disturbances to her gait and speech, for example, one doctor eventually thought to test her for HAT, but only after five days of presumptive treatment for bacterial meningitis and investigations for malaria. Having never treated or even seen a case of HAT before, the doctor had no embodied syndromic knowledge to draw on and said she was unsure if the case could be due to HAT, but she did recall being trained in hospital lab and treatment protocols several months previously, which gave her the idea. This case demonstrated that when a diagnoser’s HAT reflexes were weak because of a lack of direct experience with the disease and its symptoms, simply knowing that a HAT test was available could be sufficient to prompt HAT testing in contexts when diagnosers had run out of options.

### 3.3. Serendipitous Detection

In patient stories featuring serendipity, lay people and HCWs expressed that they had never purposely thought to test for HAT. Rather, it seemed to happen to them in a stroke of luck.

In one story, a lab technician told how he came across trypanosomes while examining a blood film of a man experiencing mood changes, confusion and fevers. The patient had come with a note from a clinician requesting simply that a film be done to identify “any infections that needed to be treated”. Not looking for trypanosomes specifically and not using any of the specialised HAT tests normally employed, it was remarkable that he found the parasites by chance. *Gambiense* trypanosomes normally occur at such low levels in the bloodstream that even the most skilled technicians often cannot confirm infections this way; it would be even more difficult for someone not specifically looking for them.

In another story, a patient recalled that he came to Nimule Hospital because it was the biggest hospital he knew of and so must have the biggest selection of tests. With what he thought of as severe malaria symptoms that were not caused by malaria, he knew he wanted to get tested for as many other diseases as possible. He went straight to the hospital lab and joined a queue of people waiting to be tested for HAT. Serendipity seemed to feature in this part of the decision. HAT testing was the only lab service for which patients could self-initiate testing. If he had joined any other queue, lab staff would have sent him to the outpatient department to seek a test order from a clinician who might not have thought to test him for HAT.

In neither story was there a true ‘diagnoser’ to whom a syndromic HAT suspicion could be attributed or even a ‘decision-maker’ that actively pursued a HAT test as in the previous typologies. While the HCW who ordered the blood film possessed suspicion for a range of diseases, he did not actually request a HAT test and the lab technician who detected the parasite did not know he was performing a ‘HAT test’ until he had made the diagnosis. Likewise, having recently moved to Nimule from a non-endemic area, the patient wanting lots of tests had no prior knowledge of HAT or the HAT service. The man he queued behind simply told the patient what test he was waiting for and recommended the patient try this test first because otherwise he would have to wait much longer to be seen in the outpatient department.

Nevertheless, there were HAT diagnostic skills involved: the remarkably practiced eye of the lab technician and the navigational knowledge of the man in the queue who also appreciated the option of a self-testing service and shared it willingly. What enabled these peoples’ skills to be practiced was other peoples’ openness to the possibilities of the medical diagnostic process to arrive at a cause for the illness, whether this involved placing trust in a big hospital to have a test that could help or trusting a lab technician to identify any infections that needed to be treated. Such expertise was thus not only embodied in the individuals involved, it was embedded in the routines, practices and habits of the hospital lab and its relationships to the HCWs and lay people who used it.

## 4. Discussion

### 4.1. Extending the Reflex Metaphor

The importance of embodied knowledge is rarely discussed in public health. Through the idiom of the diagnostic ‘reflex’, a discourse about embodied knowledge has emerged in the HAT field related to the nature of expertise in the context of disease elimination. While specific to the HAT field, the idea of a reflex nevertheless encapsulates a problem all disease control programmes must address: how to translate abstract diagnostic knowledge into embodied practice.

The vocabulary of the reflex helps us think about how diagnosis happens in places with limited health infrastructure where people rely on their senses and work with the biomedical and laboratory resources available to them to successfully diagnose disease. In discourses about the HAT reflex, curricular training in diagnostic protocols is insufficient to make an expert. Rather, diagnostic expertise develops through HCWs’ routine exposures to HAT. This contact acts as stimulation, allowing HCWs to put their knowledge into practice and continually exercise or hone their reflexes. The anatomical origins of the term hints at what else experts know about HAT expertise: that it is something contained within their minds and bodies, accrued through the expert’s personal material and social experiences with disease, instantly available to be used by them in context. 

Several observations from our study in Nimule help refine our understanding of the reflex metaphor and suggest how it can be extended. First, HCWs here used embodied knowledge to recognise HAT often in the medical ward and in domestic settings outside of working hours but rarely in busy hospital outpatient encounters. This suggested that as much as a HAT reflex is habitual and can appear to happen quickly, time and space help clinicians to respond to a sense that something is abnormal and emphasises the fundamentally creative nature of this reflex [17].

Second, our data suggests that in addition to the minds and bodies of HCWs, a diagnostic reflex is also present in lay people who are familiar with HAT. In Nimule, not only did lay people play an (independent) role in forming diagnoses, they used similar practices as HCWs to do so. Sociologists argue that for self-diagnosis to happen, medical knowledge must escape the walls of a hospital and the bounds of medical authority [17]. In the case of HAT, one could argue that the intermittent accessibility of HAT services through active screening means that successful case recognition through ‘passive’ means has often relied on the agency of lay people and HAT diagnostic knowledge has never been the sole domain of medical experts. Control programmes should thus avoid misattributing the substantial diagnostic work done by lay people to HCWs; otherwise we risk limiting our expectations about how lay people can and should participate in HAT elimination.

Third, given that expertise can be shared through creation of organizational and social norms [18], HAT diagnosis also appears to happen through the activation of expert knowledge that exists outside of expert bodies, stored in the routine practices of HCWs’ and lay peoples’ interactions with the hospital lab. Like a neural impulse that becomes apparent as it prompts action while travelling along a network or system of neural connections, our data has encouraged us to think of the HAT reflex as something that is distributed within a healthcare *system*.

### 4.2. Non-Expert Forms of Practice

According to health systems thinking, people of all sorts—whether HCWs or lay—are at the centre of health systems, mediating them and helping drive them, though they draw on different sorts of experience and operate from different perspectives [51]. Lay people can thus be beneficiaries, advocates, patients, healthcare seekers, or diagnosers. Focusing on the variety of diagnostic practices of lay people helped us identify alternative routes to successful case detection through the system, not captured in expert discourses.

In Nimule, pragmatic testing was a form of non-expert diagnostic practice that led to HAT detection and which, while also sometimes used by HCWs, was particularly suited to lay peoples’ needs. In this type of practice, HAT tests were used in ways that seemed casual or tentative—undertaken, at the end of a process rather than in a tenacious, concerted effort to confirm syndromic suspicions as in the expert reflex or to rule out a potentially life-threatening disease as in some HCWs’ approaches to their duty of care [52]. In their first steps in the pragmatic testing process, diagnosers did not even consider HAT as a potential diagnosis or test for it. Pragmatic testing was not based on personal experience with the syndromic presentation of HAT, rather it was based on practical knowledge of available tests. In many impoverished healthcare settings, “[diagnostic] uncertainty is so perpetual as to become banal” [48] (p.817). Diagnosers therefore suspend action towards diagnostic ‘closure’ or categorisation and instead focus on what is practical in the face of imperfect knowledge. In Nimule, for both lay people and HCWs at the hospital, progressive testing through a discrete number of infectious diseases options, including HAT, was an accessible way of solving healthcare problems and engaging with the opportunities of laboratory and biomedical resources. Diagnosis, after all, is not only ‘for’ the abstract categorization of states of ill health; it is also a means to access treatment [36]. In this circumstance, peoples’ pursuit of tests for treatment also had the beneficial effect of leading to case detection.

In our data, the more embodied the HAT reflex was, the more certain people were of a HAT diagnosis based on syndromic criteria. Patients in this case series were mostly in stage 2, so it generally took patients in Nimule a long time to achieve a correct diagnosis of HAT (an average of 9.7 months (median 6) on 2.8 visits seeking healthcare before being tested for HAT, Supplemental File S4). While our study was not designed to explore reasons for diagnostic delay or opportunities for missed diagnosis, it may be reasonable to assume that patients detected later had later or less contact with an expert HAT diagnoser. Descriptive quantitative analysis of our small sample supports this idea, suggesting that when a diagnoser with an embodied syndromic reflex was involved, cases tended to be identified more quickly, whether this was a HCW or a lay person (an average of 6.1 months on 1.8 visits for people identified using syndromic suspicion compared to 9.8 months on 3.2 visits using a pragmatic testing approach, Supplemental File S4).

Case detection not only happened along a *decreasing* ‘index’ or gradient of certainty, however. We also saw it happening along an *increasing* gradient of serendipity, given that the more open and familiar people were with the hospital systems, the more chances they had to arrive at a diagnosis by surprise. While patients and other people involved in detection of serendipitous cases saw the un-sought diagnosis as unexpected good fortune, dismissing such events as simply the result of chance underappreciates how interpreting serendipity as a “mix of accident and sagacity” contributes to an analysis [53] (p.139). For us, given that we were seeking to uncover the implicit structures, behaviours and conditions which led to HAT diagnosis, paying attention to apparently serendipitous cases enabled us to identify unexpected ways that the HAT reflex could be seen as translated across and embedded within a system. 

### 4.3. Preparing Health Systems for HAT Elimination

Can a HAT reflex based on embodied knowledge be cultivated in contexts with less disease? As long as HAT transmission continues and at least some of the cases in a population continue to be detected, diagnostic expertise can be expected to develop, albeit at a slower rate. As suggested by HAT expert fears of the threat of elimination to their reflexes, we should nevertheless treat HAT case detection events as important opportunities for learning. Moreover, an inclusive approach to health systems development in which diagnostic learning by many different types of actor is encouraged means that many peoples’ reflexes will be primed, ready to identify the final cases of HAT for elimination.

Hospitals in endemic areas, for example, could use admitted patients to purposely—and practically—teach the symptoms of HAT to other clinicians in their facility. Patients and the lay people involved in their detection can also be given feedback on their diagnostic skills and offered opportunities to further develop their expertise through trainings or participate in volunteer HAT detection or community surveillance programmes.

HAT RDTs will also be part of the solution to maintaining people’s diagnostic reflexes, particularly if we resist seeing them as magical technology that enable us to by-pass untrusted local expertise [54]. HCWs in a primary health care context are familiar with using RDTs to diagnose other diseases and may have practical knowledge of HAT from living in endemic areas. With HAT expertise historically concentrated in hospitals, however, many of the HCWs who are being asked to use the HAT RDTs will need to do so without much embodied knowledge of the disease’s clinical presentation. HAT programmes deploying RDTs in these settings should thus ensure that supervisors use monitoring visits to discuss specific patient histories that primary care staff have thought (or think) could be HAT cases to regularly stimulate development of their HAT reflexes. Supervisors could also confidentially discuss the patient syndromic profiles of new cases found elsewhere within the endemic area.

Importantly, our observations from Nimule have also shown that other forms of diagnostic practice can still lead to HAT case detection when an embodied HAT reflex is not present. ‘Novice’ diagnosers can precipitate detection through pragmatic testing strategies and detection can happen serendipitously through the skills of experts which have been embedded in systems. Health systems should embrace all such practices not only for their contributions to detection today, but also for their potential contributions to the creation of embodied expertise for tomorrow. No matter how people arrive at HAT diagnoses, reflecting on how a lab diagnosis fits a patient’s syndromic presentation even after detection is an important part of the personal *experience* of diagnosis which helps embody diagnostic knowledge of HAT that can be used to identify new cases in the future.

To encourage use of HAT tests in pragmatic testing strategies, health systems should take care not to inadvertently exclude lay people from engaging with HAT tests on their own terms and acknowledge that HCWs and lay people alike always make decisions to test for HAT in relation to alternative diagnostic possibilities. 

Decoupling diagnosis from particular diseases is a key aim of patient-centred approaches in public health because of the way HCWs in primary care often diagnose and manage *types* of presenting symptoms or syndromes rather than pathologically-defined disease [55]. As in some other elimination programmes for neglected tropical skin diseases [56], HCWs in HAT elimination settings should be trained to consider a broad differential diagnosis when confronted with patient presentations that fit both typical and atypical presentations of HAT. How best to conceptualise HAT diagnosis in relation to HCWs’ existing engagements with presenting syndromes such as neurological disorders, psychiatric problems or persistent fevers, however, remains underexplored in discussions of HAT integration and the HAT reflex [12,57,58] (see also the range of medical, social and other conditions patients in our study were diagnosed with before HAT detection, Supplemental File S5).

We can also imagine situations where well-equipped systems create the possibility for serendipity. In our data, the conditions that enabled serendipitous detection involved a well-resourced laboratory with skilled staff offering a range of tests as well as a highly-engaged patient population. The lively engagement of lay people with Nimule Hospital’s HAT service related to the easy accessibility of HAT screening through the self-referral option at the lab. Not only did people enjoy bypassing outpatient systems to access it, its popularity created a physical presence in outpatient areas of the hospital through the regular formation of queues. Just as crowds and generators involved in active screening draw people in to test for HAT, the queues associated with passive screening in Nimule contributed to the test’s—and therefore the disease’s—visibility. As substantial things, diagnostic tests, like medicines, help make illnesses tangible and communicable in the collective imaginary by creating an aura of facticity [59]. RDT deployments should thus consider where, when and how we want to stimulate people’s diagnostic reflexes in a health system [60].

As we approach HAT elimination and cases of disease become more rare, our HAT diagnostic reflexes may indeed slacken as experts fear. As a living entity, however, diagnostic expertise is born and grows through practice. Treating HAT detection events as important learning opportunities and ensuring multidimensional access to HAT tests will thus be vital to provide HCWs and lay people with opportunities to practice HAT diagnosis and develop their reflexes for elimination.

## Figures and Tables

**Table 1:** Case detection typologies assigned according to the category of diagnoser, for 32 human African trypanosomiasis (HAT) patients.

Case Detection Typology	HAT Test Initiated By	
	Lay Person (# Patients)	Healthcare Worker (# Patients)
Syndromic suspicion	9	9
Pragmatic testing	8	2
Mixed (syndromic; pragmatic)	1	0
Serendipitous detection	1	2
Total	19	13

## Supplementary Materials

The following are available online at https://www.mdpi.com/2414-6366/5/2/52/s1, Supplementary File S1: Laboratory algorithm used to screen for and diagnose cases of HAT in Nimule Hospital, Supplementary File S2: Patient typology classifications, Supplementary File S3: Interview and analysis process, Supplementary File S4: Patient healthcare-seeking characteristics, Supplementary File S5: Alternative diagnoses given to patients.
