# Supplementary material for: Understanding the Role of the Diagnostic ‘Reflex’ in the Elimination of Human African Trypanosomiasis
Source: Trop Med Infect Dis. 2020 Apr 1;5(2):52. doi: 10.3390/tropicalmed5020052 (PMC7345297; doi:10.3390/tropicalmed5020052)
Supplement: Supplementary file 1 [file tropicalmed-05-00052-s001.zip › tropicalmed-676006 1st/Submitted files/Supp file S1 - Screening algorithm (no change).docx]

**Supplementary file S1. Laboratory algorithm used to screen for and diagnose cases of HAT in Nimule Hospital**

Patients’ blood was initially serologically screened for evidence of immunological contact with trypanosomes using the CATT test which requires a small drop of blood from the fingertip. Patients who screened positive underwent further testing to confirm active infection using larger volumes of blood, lymph and/or cerebrospinal fluid (CSF) or to increase confidence in a diagnosis based only on serological evidence using CATT with increasing dilutions of blood serum. A HAT case was defined as (i) positive microscopy on lymph or CSF directly or on blood using hematocrit concentration (Woo test) or (ii) positive CATT on blood serum at dilution 1:16 with no parasites detected. Patients with inconclusive test results (positive CATT up to dilution 1:8 with no parasites detected) were invited to present for re-testing after 3 months. After treatment, patients were followed up for two years, at which point they were declared cured, if no parasites were found in any body fluid. The CATT was not used for patients who had previously been treated for HAT, as immunological responses can persist for years after cure.
