# Supplementary material for: Understanding the Role of the Diagnostic ‘Reflex’ in the Elimination of Human African Trypanosomiasis
Source: Trop Med Infect Dis. 2020 Apr 1;5(2):52. doi: 10.3390/tropicalmed5020052 (PMC7345297; doi:10.3390/tropicalmed5020052)
Supplement: Supplementary file 1 [file tropicalmed-05-00052-s001.zip › tropicalmed-676006 1st/Submitted files/Supp file S3 - Interviews and analysis process (no change).docx]

**Supplementary file S2. Interview and analysis process**

To our knowledge, this was the first study to collect information on immediate events leading up to HAT testing in near real-time, using very short recall periods and triangulation of data from multiple lay and HCW perspectives.

Patients within active periods of health seeking and suffering traumatic illness experiences often find it difficult to recall and explain their patterns of thought in a coherent and consistent way (Dean, Tolhurst et al. 2019). HCWs also face difficulties narrating their sense-making processes; when faced with an unfamiliar or difficult diagnostic situation it is tempting for HCWs to adopt overly simplistic narrative scripts which capture the most obvious, but not necessarily the most cogent, narrative (Davenport 2011). We addressed these anticipated problems in three main ways: by collecting as much data as possible close to the time of successful diagnosis, by collecting data from multiple sources, and by sometimes interpreting apparent contradictions in patient stories as each contributing to the sense-making process, recognising that decision-making for health-related behaviours is often complex, capable of being driven by more than one idea or different ideas in different contexts (Allen 1991).

Interviews were conducted in local languages using a team of five assistants fluent in Madi or Dinka, Arabic and English who provided both linguistic and cultural interpretation. Interviews were audio-recorded and fully transcribed then translated into English for analysis. All field notes and transcripts were discussed by the research team as they were produced to inform subsequent research activities. They were then coded line by line following the constant comparative method to test the limits of emergent theories before declaring theoretical learning on themes saturated (Green and Thorogood 2004). After the first ten interviews were conducted, a master contact summary form was created and completed for each case to reflect key information from all data sources. This included participants' recorded words to retain information about the context as well as interpretive observations by the research team that rationalised multiple versions of events or highlighted evidence suggesting multiple behaviours contributed to successful detection in a single case. Key information was then carried over to a master matrix to compare experiences across cases where typologies were assigned and revised in relation to evolving definitions of the typologies themselves and the categories of people who suggested the diagnosis.

References:

Allen, T. (1991). The quest for therapy in Moyo District. Changing Uganda: The Dilemmas of Structural Adjustment and Revolutionary Change. H. Bernt-Hansen and M. Twaddle. London, James Currey**:** 149-161.

Davenport, N. (2011). "Medical residents' use of narrative templates in storytelling and diagnosis." Social Science & Medicine **73**: 873-881.

Dean, L., R. Tolhurst, G. Nallo, K. Kollie, A. Bettee and S. Theobald (2019). "Neglected tropical disease as a ‘biographical disruption’: Listening to the narratives of affected persons to develop integrated people centred care in Liberia." PLOS Neglected Tropical Diseases **13**(9): e0007710.

Green, J. and N. Thorogood (2004). Qualitative methods for health research. London, Sage.
