# Supplementary material for: Understanding the Role of the Diagnostic ‘Reflex’ in the Elimination of Human African Trypanosomiasis
Source: Trop Med Infect Dis. 2020 Apr 1;5(2):52. doi: 10.3390/tropicalmed5020052 (PMC7345297; doi:10.3390/tropicalmed5020052)
Supplement: Supplementary file 1 [file tropicalmed-05-00052-s001.zip › tropicalmed-676006 1st/Submitted files/Supp file S4 - Treatment-seeking characteristics (revised).docx]

**Supplementary file S4. Patient healthcare-seeking characteristics**

Summary statistics were calculated to compare characteristics of the patient sample in relation to the categories of their diagnoser and their case detection typology. Descriptions of how patient characteristics were defined are available in Table 1.

Table 1. Construction of variables describing the patient sample

| **Patient variable** | **Variable attributes** | **Data source** | **Details of variable construction** |
| --- | --- | --- | --- |
| Gender | Male, female | Patient files |  |
| Age | Measured in years | Patient files |  |
| Ethnicity | Dinka, Madi, other ethnic group | Patient interview |  |
| Disease stage | 1, 2 | Patient files |  |
| Symptoms of advanced disease | KI<90%, ataxia, visual or auditory hallucinations, convulsions | Patient files | Using the hospital's HAT patient admission chart which prompts clinicians to examine 32 aspects of patient health before beginning treatment, four symptoms judged to be among the most objectively verifiable and characteristic of advanced disease were selected with the assistance of HAT programme medical staff. |
| Care-seeking visits before HAT test initiated | Measured in numbers of visits | Patient interview | A care-seeking visit was defined as any time a patient visited a health facility, drug shop or traditional medicine practitioner for advice about any of the HAT symptoms they reported during admission for HAT treatment. Patients’ own judgement on how long a symptom had been consistently bothering them was used to classify whether healthcare visits where treatments were given for alternative diagnoses (such as ‘malaria’) were relevant to their current illness. The hospital visit which led to HAT testing was only classified as a healthcare ‘visit’ if the patient was presenting for a HCW’s opinion on his/her symptoms, as opposed to self-referral for a HAT test. |
| Time spent seeking care | Measured in months | Patient interview | The difference between the date of the patient's first care-seeking visit and the date of the patient's HAT test classifying them as a case or a serological suspect to be actively followed by the HAT programme. This variable was chosen rather than the time since symptom onset because patients’ personal health books could often be used to specify the timing of visits in their accounts. When no record was available and patients verbally estimated a time range, the shortest time was used. |

Patients spent an average of 9.7 months (median 6) on 2.8 visits seeking healthcare before being tested for HAT (Table 2). Patients spent roughly the same amount of time seeking care on a similar number of visits, no matter which type of person initiated the test (10.1 months and 2.8 visits for patients detected by lay people versus 9.2 months and 2.9 visits for those detected by HCWs). Neither type of diagnoser was more involved in detecting patients with advanced symptoms of disease apart from patients with hallucinations, for whom HCWs more often initiated HAT testing (4/5 patients).

Patients identified using a HAT reflex through the syndromic suspicion typology, however, spent less time seeking care than those identified through pragmatic testing (an average of 6.1 months on 1.8 visits compared to 9.8 months on 3.2 visits), and patients had more advanced symptoms (7/9 patients with KI<90%, 5/6 with ataxia and 4/5 with convulsions).

Females in the series were more often detected after testing that was initiated by a lay person than a HCW (9/14 or 64% of females) and via the syndromic suspicion typology (9/13 females). The patients detected by HCWs were slightly younger than those detected by lay people (the median age tested by HCWs was 22 years compared to 30 by lay people), though children ≤14 years were detected as often by lay people as HCWs (2 children detected by each type of diagnoser). The majority of Dinka patients (13/16 or 81%) were detected by lay people, while HCWs were more often involved in detecting Madi patients (6/11) and those from other ethnic groups (4/5).

Table 2. Demographic, disease and healthcare-seeking characteristics of 32 HAT patients, according to the type of diagnoser who initiated HAT testing and their diagnostic typology

|  |  | **Type of diagnoser** | | | **Diagnostic typology** | | |
| --- | --- | --- | --- | --- | --- | --- | --- |
| **Variable** | **Attributes** | **Total**  **(32 patients)** | **Lay person**  **(19 patients)** | **HCW**  **(13 patients)** | **Total**  **(28 patients)** | **Syndromic suspicion**  **(18 patients)** | **Pragmatic testing**  **(10 patients)** |
| Gender | Male | 18 | 10 (55.6%) | 8 (44.4%) | 15 | 9 (60.0%) | 6 (40.0%) |
|  | Female | 14 | 9 (64.3%) | 5 (35.7%) | 13 | 9 (69.2%) | 4 (30.8%) |
| Age | Mean (in years) | 27.8 | 28.8 | 25.3 | 26.3 | 26.3 | 26.1 |
|  | Median (range) (in years) | 28 (11-65) | 30 (11-65) | 22 (11-44) | 28.5 (11-44) | 29 (11-41) | 23.5 (11-44) |
| Ethnicity | Dinka | 16 | 13 (81.3%) | 3 (18.8%) | 13 | 9 (69.2%) | 4 (30.8%) |
|  | Madi | 11 | 5 (45.5%) | 6 (54.5%) | 10 | 5 (50.0%) | 5 (50.0%) |
|  | Other | 5 | 1 (20.0%) | 4 (80.0%) | 5 | 4 (80.0%) | 1 (20.0%) |
| Disease stage | 1 | 2 | 2 (100.0%) | 0 (0.0%) | 2 | 1 (50.0%) | 1 (50.0%) |
|  | 2 | 27 | 17 (63.0%) | 10 (37.0%) | 23 | 15 (65.2%) | 8 (34.8%) |
|  | Unknown | 3 | 0 (0.0%) | 3 (100.0%) | 3 | 2 (66.7%) | 1 (33.3%) |
| Symptoms of advanced disease | KI <90%^1^ | 12 | 6 (50.0%) | 6 (50.0%) | 9 | 7 (77.8%) | 2 (22.2%) |
|  | Ataxia | 7 | 4 (57.1%) | 3 (42.9%) | 6 | 5 (83.3%) | 1 (16.7%) |
|  | Hallucinations | 5 | 1 (20.0%) | 4 (80.0%) | 3 | 2 (66.7%) | 1 (33.3%) |
|  | Convulsions | 5 | 2 (40.0%) | 3 (60.0%) | 5 | 4 (80.0%) | 1 (20.0%) |
| Time spent seeking care^2^ | Mean (in months) | 9.7 | 10.1 | 9.2 | 7.5 | 6.1 | 9.8 |
|  | Median (range) (in months) | 6 (0-36) | 7 (0-36) | 6 (0-36) | 5 (0-36) | 5 (0-24) | 5.5 (2-36) |
| Care-seeking visits before HAT test initiated^2^ | Mean (number of visits) | 2.8 | 2.8 | 2.9 | 2.3 | 1.8 | 3.2 |
|  | Median (range) (number of visits) | 3 (0-12) | 2.5 (0-12) | 3 (0-6) | 2 (0-6) | 2 (0-6) | 3 (1-5) |

^1^ KI: Karnofsky’s Index which measure's patient functionality. A score of 90-100% indicates minor symptoms or no evidence of disease. A score of 80% corresponds to patient accomplishes “normal activities with effort”, 50% to “needs constant follow up and frequent care”, 10% to “moribund, death imminent”. Data included 3 patients who died before assessment, therefore KI on admission presumed to be <90%.

^2^ The time spent seeking care and the number of visits was unknown for 1 patient detected after testing via lay-initiated syndromic suspicion. Patients classified as being detected through mixed and serendipitous detection typologies were excluded from typology-specific analyses.
