# Supplementary material for: Understanding the Role of the Diagnostic ‘Reflex’ in the Elimination of Human African Trypanosomiasis
Source: Trop Med Infect Dis. 2020 Apr 1;5(2):52. doi: 10.3390/tropicalmed5020052 (PMC7345297; doi:10.3390/tropicalmed5020052)
Supplement: Supplementary file 1 [file tropicalmed-05-00052-s001.zip › tropicalmed-676006 1st/Submitted files/Supp file S5 - Alternative diagnoses considered (new).docx]

**Supplementary file S5. Alternative diagnoses given to 32 HAT patients, in relation to their presenting symptoms**

The table below lists all alternative diagnoses mentioned during patient treatment-seeking narratives, given by anyone they consulted for help with HAT-like symptoms

| **Diagnostic category** | **# Patients** | **Alternative diagnosis** | **To explain HAT symptoms** |
| --- | --- | --- | --- |
| **Infections** | 19 | Malaria | Fever, pains, weakness, weight loss, convulsions, hemiplegia, paraesthesia, difficulty speaking, difficulty walking, mental confusion, hallucinations, aggression, abortion |
|  | 16 | Typhoid | Fever, pains, weakness, weight loss, convulsions, hemiplegia, difficulty speaking, difficulty walking, mental confusion, hallucinations, aggression |
|  | 3 | Brucellosis | Fever, confusion, paraesthesia |
|  | 2 | Bacteria (undefined) | Fever and body pains, painful lymph nodes |
|  | 2 | HIV/AIDS | Fever, weight loss, mental confusion, aggression |
|  | 1 | Onchocerciasis | Itching |
|  | 2 | Pelvic inflammatory disease, syphilis | Abortion, hemiplegia, back pain |
|  | 2 | Schistosomiasis | Fever, abdominal pains |
|  | 1 | Meningitis | Convulsions, difficulty walking, difficulty speaking |
|  | 1 | Tuberculosis | Fever, weakness, weight loss |
| **Social explanation** | 4 | Drunkenness | Convulsions, mental confusion, aggression |
|  | 4 | Poisoning (magical) | Pains, paraesthesia, convulsions, hemiplegia |
|  | 5 | Spirit of death, vengeful spirit (ceni) or spirit of relative | Convulsions, hallucinations, nightmares |
|  | 1 | Witch doctor's curse | Hemiplegia, pains |
|  | 1 | Too much work | Back pain |
|  | 1 | Marijuana use | Confusion, aggression |
|  | 1 | Bad wife, disobeying husband | Mental confusion/excessive talking |
|  | 1 | Being a first-born child | Mental confusion |
|  | 1 | Football injury | Back pain |
|  | 1 | Soldier-type behaviour | Confusion, aggression |
|  | 1 | Mixing herbs and hospital medicine | Abortion |
| **Unidentified psychiatric diagnosis** | 4 | Psychosis, schizophrenia, hysteria or 'organic psychiatric symptoms' | Mental confusion |
|  | 1 | Depression | Mental confusion |
|  | 1 | Madness | Mental confusion, aggression |
|  | 1 | Anti-psychotic medicine side-effects | Excessive sleeping, intermittency of mental confusion |
| **Other conditions** | 3 | Pregnancy-induced weakness | Weakness, hemiplegia |
|  | 1 | Adi (traditional Madi disease) | Headache, neck pains with nose-bleeding |
|  | 2 | Goitre | Convulsions, weight loss, weakness, pains |
|  | 1 | Arthritis | Paraesthesia (prickling sensation in skin), pain, hemiplegia |
|  | 1 | Diabetes | Paraesthesia |
|  | 1 | Iron deficiency | Weakness, hemiplegia |
|  | 1 | Infection from dirty water | Itching |
